# Impact of secreted glucanases upon the cell surface and fitness of *Candida albicans* during colonisation and infection

**DOI:** 10.1016/j.tcsw.2024.100128

**Published:** 2024-06-04

**Authors:** Qinxi Ma, Arnab Pradhan, Ian Leaves, Emer Hickey, Elena Roselletti, Ivy Dambuza, Daniel E. Larcombe, Leandro Jose de Assis, Duncan Wilson, Lars P. Erwig, Mihai G. Netea, Delma S. Childers, Gordon D. Brown, Neil A.R. Gow, Alistair J.P. Brown

**Affiliations:** a MRC Centre for Medical Mycology, Geoffrey Pope Building, University of Exeter, Exeter EX4 4QD, UK; b Institute of Medical Sciences, University of Aberdeen, Foresterhill, Aberdeen AB25 2ZD, UK; c Department of Internal Medicine and Radboud Center for Infectious Diseases, Radboud University Medical Center, Nijmegen, the Netherlands; d Department for Immunology & Metabolism, Life and Medical Sciences Institute (LIMES), University of Bonn, 53115 Bonn, Germany

**Keywords:** *Candida albicans*, Cell wall, Xog1 exoglucanase, Eng1 endoglucanase, β-glucan shaving, Gut colonisation, Virulence

## Abstract

Host recognition of the pathogen-associated molecular pattern (PAMP), β-1,3-glucan, plays a major role in antifungal immunity. β-1,3-glucan is an essential component of the inner cell wall of the opportunistic pathogen *Candida albicans*. Most β-1,3-glucan is shielded by the outer cell wall layer of mannan fibrils, but some can become exposed at the cell surface. In response to host signals such as lactate, *C. albicans* shaves the exposed β-1,3-glucan from its cell surface, thereby reducing the ability of innate immune cells to recognise and kill the fungus. We have used sets of barcoded *xog1* and *eng1* mutants to compare the impacts of the secreted β-glucanases Xog1 and Eng1 upon *C. albicans in vitro* and *in vivo*. Flow cytometry of Fc-dectin-1-stained strains revealed that Eng1 plays the greater role in lactate-induced β-1,3-glucan masking. Transmission electron microscopy and stress assays showed that neither Eng1 nor Xog1 are essential for cell wall maintenance, but the inactivation of either enzyme compromised fungal adhesion to gut and vaginal epithelial cells. Competitive barcode sequencing suggested that neither Eng1 nor Xog1 strongly influence *C. albicans* fitness during systemic infection or vaginal colonisation in mice. However, the deletion of *XOG1* enhanced *C. albicans* fitness during gut colonisation. We conclude that both Eng1 and Xog1 exert subtle effects on the *C. albicans* cell surface that influence fungal adhesion to host cells and that affect fungal colonisation in certain host niches.

## 1 Introduction

Innate immune cells provide an early line of defence against microbial infection. Many pathogenic microbes are kept at bay through recognition of their pathogen-associated molecular patterns (PAMPs) by pattern recognition receptors (PRRs) expressed by host cells (Dambuza et al., 2017, Erwig and Gow, 2016, Lionakis and Levitz, 2018, Pathakumari et al., 2020). This PRR-PAMP-based recognition is critical for the activation of antimicrobial killing and clearance mechanisms, the recruitment of immune cells to the site of infection, and the induction of innate and adaptive immunity (Brown, 2011, Domínguez-Andrés et al., 2023, Netea et al., 2017).

The opportunistic fungal pathogen *Candida albicans,* causes debilitating mucosal infections such as oral and vulvovaginal candidiasis, particularly in those individuals with compromised T cell immunity (Davidson et al., 2018, Vila et al., 2020, Yano et al., 2019). *C. albicans* also causes life-threatening systemic disease, often in patients with severe neutropenia (Lionakis and Levitz, 2018, Pappas et al., 2018)*.* In immunocompetent individuals, the recognition of PAMPs such as β-1,3-glucan by innate immune cells plays a critical role in anti-*Candida* immunity (Bojang et al., 2021, Erwig and Gow, 2016, Netea et al., 2006). β-1,3-glucan is an essential component of the *C. albicans* cell wall (Childers et al., 2020b, Douglas et al., 1997, Gow and Lenardon, 2023), and plays a major role in antifungal immunity (Brown and Gordon, 2001, Ferwerda et al., 2009, Iliev et al., 2012, Lowman et al., 2014) being recognised by multiple PRRs including dectin-1, CD36 and NLRP3 (Dambuza et al., 2017, Erwig and Gow, 2016). Most β-1,3-glucan is located in the inner layer of the *C. albicans* cell wall, and hence is essentially shielded from immune recognition by the outer cell wall layer of mannan fibrils (Lenardon et al., 2020). However, some β-1,3-glucan can become exposed at the *C. albicans* cell surface, for example at new bud scars on mother cells and at punctate features on both mother and daughter cells (de Assis et al., 2022). These sites of β-1,3-glucan exposure are targets for innate immune cell recognition and phagocytosis (de Assis et al., 2022).

However, *C. albicans* is carried as a relatively harmless commensal in the oral cavity and gastrointestinal tract of many immunocompetent individuals (Gouba and Drancourt, 2015, Hallen-Adams and Suhr, 2017, Kumamoto et al., 2020, Nash et al., 2017), suggesting that this fungus can evade or avoid host immunity. Indeed, *C. albicans* exploits a range of immune evasion strategies (Marcos et al., 2016, Underhill, 2007) one of which involves the shaving of exposed β-1,3-glucan from the cell surface (Ballou et al., 2016, Childers et al., 2020a, Pradhan et al., 2018, Pradhan et al., 2019, Pradhan et al., 2021). In response to specific host signals, including exposure to lactate, neutral ambient pHs, hypoxia or iron depletion, *C. albicans* reduces the exposure of β-1,3-glucan at its cell surface, in part by shaving, and this reduces the ability of innate immune cells to recognise the fungus (Ballou et al., 2016, Childers et al., 2020a, Pradhan et al., 2018, Pradhan et al., 2019, Sherrington et al., 2017).

Both Xog1 (Childers et al., 2020a) and Eng1 (Yang et al., 2022) have been implicated in β-1,3-glucan shaving. *XOG1* encodes the major exoglucanase in *C. albicans* and the inactivation of this gene was found to significantly reduce fungal burdens in the brain, but not in the kidney, in a mouse model of systemic infection (Del Mar González et al., 1997). In *Saccharomyces cerevisiae*, secretion of the Eng1 endoglucanase by daughter cells promotes their separation from the mother cell by degrading β-glucan at the septal junction, and Eng1 is thought to play a similar role in *C. albicans* (Yang et al., 2022). Consequently, in *C. albicans*, new bud scars on mother cells expose β-1,3-glucan whereas those on daughters do not (de Assis et al., 2022) and *ENG1* deletion leads to increased β-1,3-glucan exposure (Yang et al., 2022). Significantly, increased levels of both Xog1 and Eng1 are observed in the *C. albicans* secretome following exposure to lactate or hypoxia (Childers et al., 2020a). Both of these signals promote β-1,3-glucan masking (Ballou et al., 2016, Pradhan et al., 2018).

An inverse correlation has been reported between the competitive fitness of *C. albicans* in the gastrointestinal tract and the exposure of β-1,3-glucan at the fungal cell surface (Sem et al., 2016). Furthermore, hypoxia-induced β-1,3-glucan masking appears to attenuate antifungal immunity during infection (Lopes et al., 2018) and lactate exposure enhances the virulence of *C. albicans* in a murine model of disseminated candidiasis (Ene et al., 2012). Hence, we reasoned that β-1,3-glucan shaving, and the Xog1 and Eng1 β-glucanases in particular, might play a role during infection. Therefore, in this study, we have compared the impact of these glucanases upon *C. albicans* cell wall architecture, cell wall stress resistance, adherence to epithelial cells, and fitness during systemic infection and gut and vaginal colonisation.

## 2 Materials and methods

### 2.1 *C. albicans* strains and growth

The *C. albicans* strains used in this study are listed in Supplementary Table S1. To generate an isogenic set of differentially barcoded, prototrophic *C. albicans* strains (Larcombe et al., 2023) (Supplementary Table S1), *C. albicans* CAI4 (*ura3*) was transformed with CIp10-Ptet-GTW plasmids (*URA3*) carrying different barcodes (Cabral et al., 2014), integrating these plasmids at the *RPS1* locus (Brand et al., 2004, Murad et al., 2000). Homozygous *xog1* and *eng1* null mutants were made in these barcoded *C. albicans* strains using the transient CRISPR-Cas9 method as described previously (Min et al., 2016). The primers used are listed in Supplementary Table S2.

For most experiments, *C. albicans* strains were grown at 30 °C with shaking at 200 rpm in glucose-containing minimal medium (GYNB without amino acids: 2 % glucose, 0.65 % yeast nitrogen base without amino acids) (Sherman, 1991). Where specified, cells were grown instead in rich medium (YPD: 2 % glucose, 1 % yeast extract, 2 % myco-peptone) (Sherman, 1991).

### 2.2 Cell wall stress assays

*C. albicans* strains were grown overnight in 3 ml YPD broth medium at 30 °C with shaking at 200 rpm. Overnight cultures were collected by centrifugation at 6,000 x *g* at room temperature for 5 min. Cells were washed twice with 1 ml PBS and resuspended in 1 ml PBS. Each cell suspension was adjusted to an OD_600_ of 0.0625 in PBS. Strains were then serially diluted five-fold in PBS to a final OD_600_ of 0.0001. Each dilution (5 μl) was then plated onto YPD agar containing no stress, 62.5 ug/ml Congo Red, 10 mM caffeine, 0.1 ug/ml caspofungin or 0.05 ug/ml anidulafungin, and the plates were incubated at 30 °C and imaged after three days. Stress sensitivities were also examined by monitoring growth (OD_600_) of cells in liquid YPD in 96-well format.

### 2.3 Cytometric quantification of β-1,3-glucan exposure

Yeast strains were grown in GYNB overnight at 30 °C and 200 rpm. Overnight cultures were inoculated into 5 ml of fresh GYNB to an OD_600_ of 0.2 containing 0 (Glu) or 2 % lactate (Glu-Lac). After growth at 30 °C and 200 rpm for 5 h, cells were fixed overnight with 50 mM thimerosal (Sigma-Aldrich, Dorset, UK), washed with water, and stained with Fc-Dectin-1 and anti-human IgG linked to Alexafluor 488 (Life Technologies, Paisly, UK or Jackson ImmunoResearch, Ely, UK) (Ballou et al., 2016). An Attune NxT flow cytometer was used to quantify the fluorescence of 10,000 cells per sample, and the Median Fluorescence Intensity (MFI) was quantified using FlowJo v.10 software (Ballou et al., 2016). Fold changes in β-1,3-glucan exposure were calculated by dividing the MFI for cells grown in lactate (GluLac) by the MFI for the corresponding control cells grown in glucose (Glu). The mean of three independent measurements was determined for each strain.

### 2.4 Fluorescence and electron microscopy

Fluorescence microscopy was performed on *C. albicans* cells grown to exponential phase in GYNB containing 0 or 2 % lactate for 5 h at 30 °C and fixed with thimerosal (above). Cells were stained with Fc-dectin-1 and IgG-AF488 (β-1,3-glucan), and with ConA-AF647 (mannan). Cells were imaged using a DeltaVision Elite fluorescence microscope with a 100x objective and 1.40 numerical aperture. Fluorescence excitation was generated by a Lumencor LED light source and 10 µm Z-stacks of 50 images were captured by a pco.edge sCMOS camera. The 3D stacks were then deconvolved to remove out of focus light and maximum intensity projections used to create a 2D image by Image-J (version 1.54).

For transmission electron microscopy (TEM), overnight cultures grown in GYNB were harvested using polycarbonate filters, promptly transferred to aluminium planchettes (0.1 mm depth) and subjected to high-pressure freezing using a HPM Live µ, CryoCapCell (Le Kremlin-Bicêtre, France) according to published methods (McDonald and Webb, 2011). Samples were transferred under liquid nitrogen into cryotubes containing 1 % osmium tetroxide and 0.5 % glutaraldehyde in acetone, transferred onto a rotary shaker, and incubated for 3 h under dry ice. After removing the dry ice, samples were shaken for a further hour. Samples were then washed thrice in acetone for 5 min, embedded in Epon, and ultrathin sections (60 nm) prepared on pioloform-coated 100 mesh copper EM grids, using lead citrate for contrast. Samples were imaged using a JEOL 1400 JEM transmission electron microscope with a digital camera (ES1000W, Gatan, Ametek, Abingdon, UK). Sections of the *C. albicans* cell wall were selected for imaging at random at a magnification of 100 k times. At least 30 cells were imaged per condition, and the diameters of the inner and outer layers of the cell wall determined (30 measurements per cell) using the line tool in ImageJ (version 1.54).

### 2.5 Adhesion to epithelial cells

The A431 human vaginal epithelial cell line and the Caco-2 human colorectal adenocarcinoma cell line were maintained in Dulbecco’s Modified Eagle’s Medium (DMEM) GlutaMAX (Gibco, Fisher Scientific, UK) supplemented with 10 % heat-inactivated foetal bovine serum (HI-FBS) (Gibco) at 37 °C, 5 % CO_2_. For the adhesion assays, monolayers of epithelial cells were cultured to confluency in 6-well TC plates (Thermo Scientific, UK), and washed with PBS. *C. albicans* strains were grown overnight in YPD at 30 °C, harvested, washed with PBS, and cell densities adjusted to an OD_600_ of 1 in DMEM without FBS. These yeast suspensions (1 ml) were added to the washed epithelial cells, and incubated at 37 °C, 5 % CO_2_ for 1 h. Non-adherent cells were removed by washing the monolayers extensively with PBS. Then the adhered human and yeast cells were detached from the plate by scraping into 1 ml PBS. Dilutions of these cell suspensions were then plated on YPD agar to determine the CFUs for the adhered *C. albicans* cells. For each condition, three biological replicates were examined, each in duplicate.

### 2.6 Ethical statement

Animal experiments were approved by the Ethical Review Committee at the University of Exeter and performed in compliance with animal research ethical regulations under UK Home Office project licence number P79B6F297. For all mouse experiments, 6–10 week old female BALB/c mice (Charles River) were housed in ventilated cages (3–4 per cage), supplied with food and water *ad libitum*. During all studies, animals were monitored daily for clinical symptoms and weight loss. No surgical procedures were performed on animals prior to humane culling by cervical dislocation or high CO_2_.

### 2.7 Invertebrate model of systemic infection

*Galleria mellonella* infections were performed as described previously (Brennan et al., 2002, de Assis et al., 2022). *C. albicans* strains were grown overnight in GYNB at 30 °C, regrown to exponential phase in fresh GYNB for 5 h, and the cells were washed and resuspended in PBS. Each strain (about 1 x 10^5^ cells in 10 μl PBS) was injected into 20 *Galleria* larvae (UK Waxworms Ltd, Sheffield, UK). The larvae were incubated at 37 °C in the dark and their survival was quantified for seven days post-infection.

### 2.8 Murine model of systemic infection

As described previously (Larcombe et al., 2023), sets of four barcoded *C. albicans* strains (Supplementary Table S1) were selected as internal replicates for each experiment to compare directly the fitness of differentially preadapted cells during systemic infection in mice. To examine the effects of lactate exposure on *C. albicans,* four barcoded strains were pre-grown on GYNB containing 0 % or 2 % lactate for 5 h, harvested and washed in PBS, and then all eight cultures pooled in approximately equal proportions.

To examine the impacts of Xog1 and Eng1, barcoded *xog1* mutants, *eng1* mutants and wild type control strains were pre-grown overnight on GYNB, regrown in fresh GYNB for 5 h, harvested and washed in PBS, and then these twelve strains pooled in approximately equal proportions.

These pools of barcoded *C. albicans* strains (100 µl containing 7 x10^5^ cells) were then injected into the lateral tail vein of three 6–10 week-old female BALB/c mice, and the mice sacrificed after 48 h. Fungal cells from the liver, spleen, brain and kidneys were plated onto YPD for 24 h, and pooled for barcode sequencing (below). To summarise, three mice were examined, each with n = 4 barcodes as technical replicates for each condition.

### 2.9 Murine model of gut colonisation

Eight 10-week-old female BALB/c mice were given sterile water containing 2 mg/ml streptomycin (Invitrogen) and 2,000 U/ml penicillin (Invitrogen) for four days before *Candida* exposure and were maintained on antibiotic water for the duration of the study. Pools of barcoded *C. albicans* strains representing the *xog1* and *eng1* mutants and their parents were prepared as described above and brought to a concentration of 1 x 10^7^ cells/100 μl in PBS. Each mouse was gavaged with 100 μl of this pooled cell suspension to colonise their gastrointestinal tract. *C. albicans* colonisation (CFUs/g of faeces) was measured daily for each mouse, and faecal samples were collected on days 3, 7, and 11 for barcode sequencing (below). In summary, six mice were examined, each with n = 4 barcodes per genotype.

### 2.10 Murine model of vulvovaginal candidiasis

The murine model of vulvovaginal candidiasis was performed as described (Wagner et al., 2022). The 6–8 week old female BALB/c mice (Charles River) were subcutaneously administered with 0.2 mg of β-estradiol 17-valerate (Sigma) dissolved in 0.1 ml sesame oil three days prior to *C. albicans* infection, and weekly thereafter. *C. albicans xog1*, *eng1* and their wild type parental control strains were grown overnight in GYNB at 30 °C, regrown to exponential phase in fresh GYNB for 5 h, washed and resuspended in PBS at a final concentration of 2 x 10^9^ cells/ml. Each mouse was inoculated intravaginally with 10 μl of cell suspension (n = 3 mice per barcoded *C. albicans* strain) and four control mice were inoculated with 10 μl PBS alone. Fungal burdens were assessed (CFUs), and IL-1β and MIP-2 levels were assayed (below) in vaginal lavage collected seven days post-infection.

### 2.11 Barcode sequencing

Barcode sequencing was used to quantify the relative proportions of the different barcodes in mixed populations of *C. albicans* strains (Larcombe et al., 2023, Pradhan et al., 2017). Homogenised tissues, lavages or faeces were plated onto YPD, grown for 24 h at 30 °C, and genomic DNA was extracted from the pooled fungal colonies (Pradhan et al., 2017). The barcodes were amplified using universal primers, and these PCR products were purified and sequenced by the Sequencing Facility at the University of Exeter, as described previously (Larcombe et al., 2023, Pradhan et al., 2017).

### 2.12 Cytokine assays

IL-1β and MIP-2 levels in murine vaginal lavage were quantified using enzyme-linked immunosorbent assay kits (Invitrogen and R&D Biosystems, respectively) according to the manufacturer’s protocols.

### 2.13 Statistical analyses

Statistical analyses were performed in GraphPad Prism 10. At least three independent biological replicates were used to generate the data, which are expressed as means ± standard deviation. A range of statistical tests were applied, as specified, depending on the nature of the dataset. The following p-values were considered: not significant, >0.05; *, *p* < 0.05; **, *p* < 0.01; ***, *p* < 0.001; ****, *p* < 0.0001.

### 2.14 Data availability

The authors declare that the data supporting the findings of this study are available within the paper (and the accompanying Supplementary Information Files).

## 3 Results

### 3.1 Prior exposure to lactate enhances *C. albicans* fitness during systemic infection

In 2016 we reported that lactate induces β-1,3-glucan masking at the *C. albicans* cell surface (Ballou et al., 2016), and this phenotype was subsequently recapitulated by other researchers in our laboratory (Childers et al., 2020a, Pradhan et al., 2019). However, following our laboratory move from the University of Aberdeen to the University of Exeter, one of these same researchers found this robust phenotype to be apparently less strong than we had first reported (Supplementary Fig. 1). While hypoxia- and iron depletion-induced β-1,3-glucan masking remained robust after the move (Pradhan et al., 2018, Pradhan et al., 2019), lactate-induced β-1,3-glucan masking was observed in some, but not in other experiments. This suggested the existence of a confounding factor that was influencing lactate-induced β-1,3-glucan masking.

The most obvious potential confounding factor was the change from soft, slightly acidic Aberdeen water to the harder water in Exeter in the southwest of England. We tested this by comparing β-1,3-glucan exposure on *C. albicans* SC5314 cells grown in media prepared with water from our laboratories in Aberdeen and Exeter, respectively. Briefly, cells were harvested after five hours of growth on glucose in minimal medium (GYNB) either in the presence or absence of lactate (Materials and Methods). These cells were then fixed and stained with Fc-dectin-1, their β-1,3-glucan exposure quantified by flow cytometry, and the degree of β-1,3-glucan masking quantified by dividing their Median Fluorescence Intensity (MFI) in the presence of lactate by their MFI in the absence of lactate (Ballou et al., 2016, Childers et al., 2020a, Pradhan et al., 2019). This revealed that the β-1,3-glucan masking phenotype was retained in cells grown on Aberdeen water but lost with the Exeter water (Supplementary Fig. 1A). Water acidity did not appear to be the confounding factor because, if anything, adjusting the water to pH 5 compromised β-1,3-glucan masking (Supplementary Fig. 1B). Similarly, the hardness of the water did not appear to be the confounding factor, as the addition of Ca^++^ and/or Mg^++^ ions did not inhibit β-1,3-glucan masking (Supplementary Fig. 1C). The issue was not resolved by using different sources of *C. albicans* SC5314, fresh versus two-week-old plates to inoculate the pre-cultures, or different batches of Fc-dectin-1. Indeed, we were unable to identify any single factor that could account for the underlying inconsistency in lactate-induced β-1,3-glucan masking. However, we found this phenotype to be more robust when overnight precultures were inoculated with fresh *C. albicans* colonies and grown for about 15 h, and when media were freshly made with Highland Spring water (a commercial Scottish water that works well in our hands: Supplementary Fig. 1D). We moved forward on this basis.

**Fig. 1:**
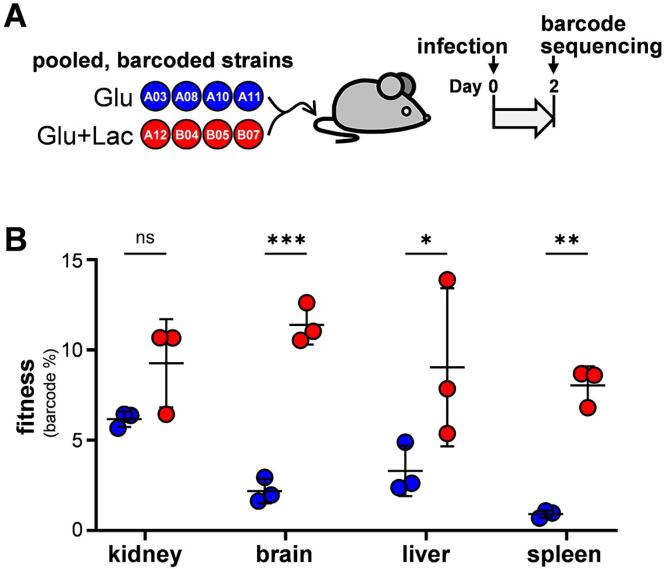
Pre-exposure to lactate enhances *C. albicans* fitness during systemic candidiasis. (A) Barcoding sequencing strategy used to compare directly the effects of pre-growing *C. albicans* cells in the presence or absence of lactate upon their fitness during the early stages of systemic infection in mice. Four isogenic barcoded strains each were grown separately in glucose-containing minimal medium (GYNB) containing 0 % or 2 % lactate: minus lactate controls, blue, Ca 2247 wt A03, Ca 2248 wt A08, Ca 2249 wt A10 and Ca 2250 wt A11; plus lactate, red, Ca 2252 wt A12, Ca 2253 wt B04, Ca 2249 wt B05 and Ca 2250 wt B07 (Supplementary Table S1). Approximately equal amounts of these cells were pooled, and this mixed population injected into the tail veins of three mice. After two days, tissues were harvested and barcode sequencing performed to determine the relative proportion of each barcoded strain in each sample. (B) The fitness of lactate-exposed cells (red) is compared to their non-exposed controls (blue) in each tissue. Each dot represents the average abundance for the replicate barcoded strains (n = 4) in one mouse (n = 3) relative to their abundance in the starting pooled inoculum. Means and standard deviations are presented; the data were analysed using two-way ANOVA (Tukey’s multiple comparisons test): ns, not significant; * *p <* 0.05; ** *p <* 0.01; *** *p <* 0.001; **** *p <* 0.0001.

Our main goal was to test the impacts of the Xog1 and Eng1 β-glucanases during infection. However, having encountered the above challenges, we first re-tested whether lactate exposure enhances the virulence of *C. albicans* during systemic infection (Ene et al., 2012)*.* To achieve this, we used eight differentially barcoded, congenic, prototrophic strains derived from the *C. albicans* clinical isolate SC5314. These strains were generated by transforming *C. albicans* CAI4 (*ura*3Δ) with differentially barcoded versions of the plasmid CIp10-PTET-GTW (*URA3*) (Cabral et al., 2014, Larcombe et al., 2023). All eight “wild type” (WT) strains were grown separately in GYNB overnight, and then four of these strains were regrown separately for five hours in GYNB (A03, A08, A10, A11), and the other four were regrown in GYNB containing lactate (A12, B04, B05, B07) (Supplementary Table S1). Approximately equal proportions of these cells were then pooled together, and this mixed population of differentially pre-adapted *C. albicans* cells was used to inoculate three mice via their lateral tail vein. We reasoned that any potential effects of lactate exposure upon *C. albicans* were likely to be most obvious during the early stages of the infection because, following inoculation, the fungus would start to adapt to the host. Therefore, the mice were sacrificed after two days, their tissues removed, fungal genomic DNA isolated, and the relative proportion of each barcoded strain measured by barcode sequencing (Materials and Methods). For *C. albicans* cells infecting the kidney, brain, liver and spleen, those that had been pre-exposed to lactate were more fit than those that were grown in the absence of lactate although, for those cells in the kidney, this difference was not statistically significant (Fig. 1). This observation reinforced our earlier finding that lactate exposure increases the virulence of *C. albicans* during systemic infection (Ene et al., 2012).

### 3.2 Eng1 contributes to lactate-induced β-1,3-glucan masking in *C. albicans*

To compare the influences of the Eng1 and Xog1 β-glucanases *in vitro* and *in vivo,* we constructed sets of congenic, barcoded, prototrophic *eng1, xog1* and wild type *C. albicans* strains using CRISPR-Cas9 technology (Materials and Methods). The *ENG1* locus was deleted in each of the barcoded *C. albicans* strains B07, B08, B11 and C10, and the *XOG1* locus was deleted in the strains C01, C08, C09 and D08, thereby generating four independent homozygous null mutants for each target locus (Supplementary Table S1). The loss of the target gene and concomitant integration of the *SAT1* marker were confirmed in each mutant by diagnostic PCR (Supplementary Fig. 2).

**Fig. 2:**
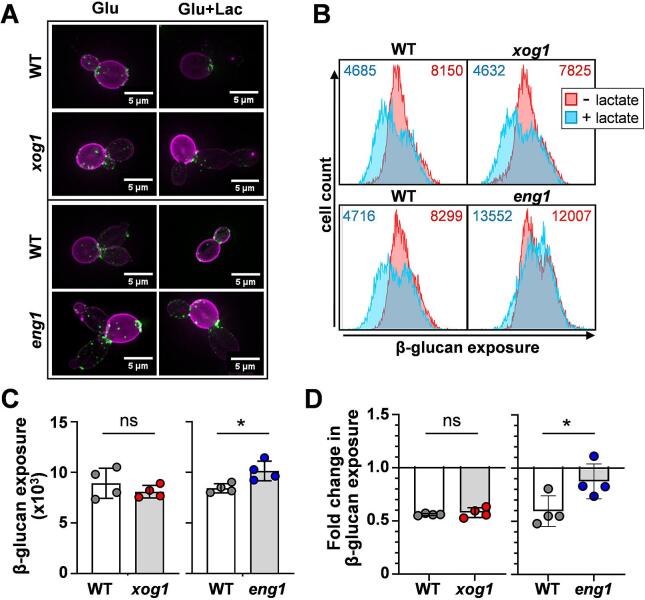
Impact of *XOG1* or *ENG1* deletion upon β-1,3-glucan exposure and masking in *C. albicans*. *C. albicans* strains were grown in GYNB containing 0 (Glu) or 2 % lactate (Glu + Lac) for 5 h, fixed, and stained with Fc-dectin-1: *xog1* B07, *xog1* B08, *xog1* B11, *xog1* C10, *eng1* C01, *eng1* C08, *eng1* C09, *eng1* D08, WT B07, WT B08, WT B11, WT C10, WT C01, WT C08, WT C09 and WT D08 (Supplementary Table S1). (A) β-1,3-glucan exposure at the cell surface was imaged by fluorescence microscopy after counterstaining the Fc-dectin-1-stained cells (green) with concanavalin A (mannan, purple). Representative images are shown. All scale bars were taken from the same images as the micrographs. (B) β-1,3-glucan exposure was quantified by flow cytometry of 10,000 cells, and representative data are presented. The MFI for each plot shown is in the top corner of each panel: pink, Glu; cyan, Glu + Lac. (C)**.** β-1,3-glucan exposure (MFI) for mutants and their corresponding parental strains after growth on glucose. (D**)** The degree of β-1,3-glucan masking (fold change in β-1,3-glucan exposure) was calculated by dividing a strain’s MFI on glucose plus lactate, by the corresponding MFI on glucose alone. In C. and D. each dot represents the mean from three independent replicates for a particular strain, and the four dots for each bar represent the four independent barcoded mutants or their corresponding parental strains: WT, grey, *xog1,* red, *eng1*, blue. Means and standard deviations for these data are shown. The data were analysed using unpaired t-tests, comparing the mutants with their parents: ns, not significant; * *p <* 0.05.

First, we showed that these *C. albicans eng1* and *xog1* mutants did not differ from wild type controls with respect to their growth in minimal medium (GYNB; Supplementary Fig. 3). Then we tested whether they displayed defects in lactate-induced β-1,3-glucan masking. Each strain was grown separately overnight in GYNB, subcultured into fresh minimal medium containing or lacking lactate, grown for five hours, the cells fixed and stained with Fc-dectin-1, and their β-1,3-glucan exposure examined by widefield fluorescence microscopy. All strains displayed the classical patterns of exposure at mother-daughter junctions, bud scars and punctate foci on their lateral cell walls (Fig. 2A), as described previously (de Assis et al., 2022).

The levels of β-1,3-glucan exposure by these strains were then quantified by flow cytometry (Fig. 2B). The β-1,3-glucan exposure displayed by the four independent *xog1* mutants was not significantly different from their four parental strains, but the *eng1* mutants exposed significantly more β-1,3-glucan (Fig. 2C). This was consistent with the observations of Yang and coworkers (Yang et al., 2022), who showed previously that Eng1 inactivation causes *C. albicans* to expose more β-1,3-glucan at their cell surface during growth in a synthetic complete medium containing amino acids. We then measured the fold decrease in β-1,3-glucan exposure induced by lactate (i.e. MFI on glucose plus lactate/MFI on glucose alone). The *eng1* mutants displayed a significant defect in lactate-induced β-1,3-glucan masking compared to their congenic parental strains (Fig. 2D), which once again was consistent with the observations of Yang and coworkers (Yang et al., 2022).

Previously, we reported that the inactivation of *XOG1* inhibits lactate-induced β-1,3-glucan masking in *C. albicans* (Childers et al., 2020a). However, unexpectedly, the four independent *xog1* mutants did not display a defect in β-1,3-glucan masking (Fig. 2D). The old *xog1* mutants were constructed by sequential deletion of each *XOG1* allele in *C. albicans* SC5314 with the *SAT1* flipper (Childers et al., 2020a, Reuss et al., 2004), whereas the new *xog1* mutants were generated using CRISPR-Cas9 in *C. albicans* CAI4 strains containing CIp10-PTET-GTW. Both sets of strains were derived from *C. albicans* SC5314. Nevertheless, it was conceivable that strain differences might account for this unexpected result (Fig. 2D). Therefore, we compared their lactate-induced β-1,3-glucan masking phenotypes directly, side-by-side. Under the refined conditions described above, neither of the two independent *xog1* mutants we constructed previously (Childers et al., 2020a) displayed an obvious defect in lactate-induced β-1,3-glucan masking (Supplementary Fig. 4). However, under these new conditions, the wild type control cells also displayed weaker masking (0.4–0.6 fold decrease in β-1,3-glucan exposure: Supplementary Fig. 4) than we had observed previously (0.6–0.8 fold decrease (Childers et al., 2020a)). Ironically, a slight decrease in β-1,3-glucan masking was observed for the four new *xog1* mutants generated in this study, but this decrease was not significant (*p* = 0.208: Supplementary Fig. 4). We conclude that Eng1 plays a more prominent role than Xog1 in lactate-induced β-1,3-glucan masking *in vitro*, at least under the conditions we examined.

### 3.3 Neither Xog1 nor Eng1 are required for the maintenance of cell wall architecture or stress resistance

**Fig. 3:**
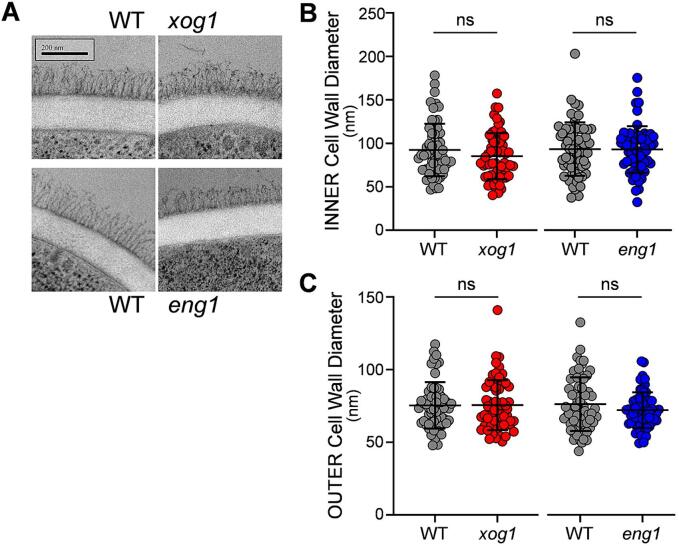
Effects of deleting *XOG1* or *ENG1* on *C. albicans* cell wall architecture. (A) The cell walls of two *C. albicans xog1* mutants and two *eng1* mutants were compared with those of their wild type controls by transmission electron microscopy of cells grown in GYNB: *xog1* B11, *xog1* C10, *eng1* C08, *eng1* D08, WT B11, WT C10, WT C08, and WT D08 (Supplementary Table S1). Representative images are shown. (B) The diameters of the inner and outer layers of the cell walls of each strain were measured using ImageJ (n = ≥30 measurements in total from about 30 randomly selected cells). The data for the two independent *xog1* and *eng1* mutants and their parents are combined in these plots, each dot representing an individual measurement: WT, grey, *xog1,* red, *eng1*, blue. Means and standard deviations are shown, and the mutants were compared with their parents using unpaired t-tests: ns, not significant.

Next, we used TEM to examine the influence of *ENG1* and *XOG1* upon cell wall architecture. The four independent *C. albicans xog1* and *eng1* mutants and their corresponding wild type parents were each grown for five hours in GYNB and immediately subjected to high-pressure freezing for TEM. All strains displayed the classical *C. albicans* cell wall bilayer: the inner layer containing chitin and β-glucan, and the outer layer of mannan fibrils (Lenardon et al., 2020). While some variation between individual cells was apparent (Fig. 3A), the mutants and their wild type parents displayed no significant differences with respect to the diameters of their inner and outer cell wall layers (Fig. 3B).

**Fig. 4:**
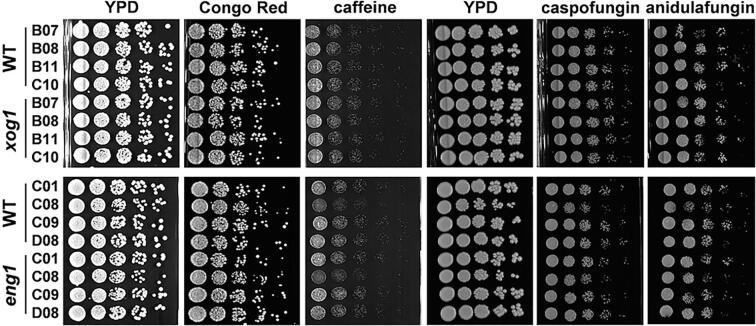
Cell wall stress resistance of *C. albicans xog1* and *eng1* mutants. The *C. albicans xog1, eng1* and wild type controls were grown overnight in YPD at 30 °C and serial dilutions plated on to YPD alone (control) or YPD containing a particular cell wall stressor: 62.5 ug/ml Congo Red; 10 mM caffeine; 0.1 ug/ml caspofungin; 0.05 ug/ml anidulafungin. Images were taken after incubating these plates at 30 °C for three days: *xog1* B07, *xog1* B08, *xog1* B11, *xog1* C10, *eng1* C01, *eng1* C08, *eng1* C09, *eng1* D08, WT B07, WT B08, WT B11, WT C10, WT C01, WT C08, WT C09 and WT D08 (Supplementary Table S1).

We then tested whether the inactivation of Eng1 or Xog1 compromises the resistance of *C. albicans* to cell wall stresses. Each of the *C. albicans xog1* and *eng1* mutants was compared to their wild type parent in plate assays to examine their resistance to Congo Red (62.5 ug/ml), caffeine (10 mM), caspofungin (0.1 ug/ml) and anidulafungin (0.05 ug/ml). No obvious differences were observed between the *xog1* or *eng1* mutants and the wild type controls on plates (Fig. 4), and only minor differences in caffeine sensitivity were observed for *xog1* cells in liquid medium (Supplementary Fig. 5). Based on these assays, we conclude that the inactivation of neither Eng1 nor Xog1 compromises the architecture or stress resistance of the *C. albicans* cell wall.

### 3.4 Impact of Xog1 and Eng1 upon the adhesion of *C. albicans* to epithelial cells

**Fig. 5:**
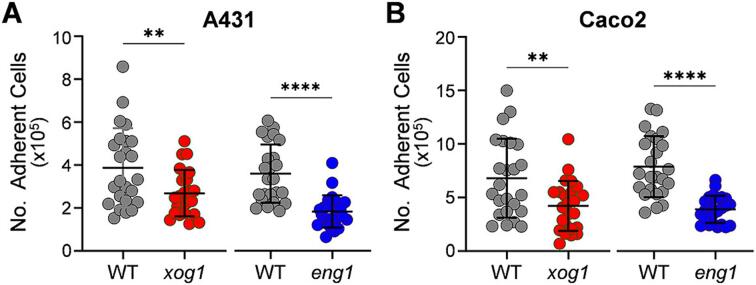
Adhesion of *C. albicans xog1* and *eng1* cells to epithelial cells. *C. albicans* strains were grown overnight, washed, resuspended in PBS and added to confluent monolayers of epithelial cells from the vagina (A**)** A431 cells) or colon (B**)** CaCo2 cells). After coincubation in for 1 h, non-adherent *C. albicans* cells were washed off, and then the adherent fungal cells measured by plating on YPD (CFUs): *xog1* B07, *xog1* B08, *xog1* B11, *xog1* C10, *eng1* C01, *eng1* C08, *eng1* C09, *eng1* D08, WT B07, WT B08, WT B11, WT C10, WT C01, WT C08, WT C09 and WT D08 (Supplementary Table S1). The data from three independent measurements, each in duplicate, for each of the four independent *xog1* and *eng1* mutants and their parents are combined in these plots, each dot representing an individual measurement: WT, grey, *xog1,* red, *eng1*, blue. Means and standard deviations are shown, and the mutants were compared with their parents using unpaired t-tests: ns, not significant; * *p <* 0.05; ** *p <* 0.01; *** *p <* 0.001; **** *p <* 0.0001.

Next, we compared the effects of deleting *XOG1* or *ENG1* upon adhesion to two different cultured epithelial cell lines: CaCo2 cells originate from the colon; and A431 cells which have different reported origins but are often used to represent the vaginal mucosa (Kohli et al., 2014). All four of the *xog1* and *eng1* mutants and their corresponding isogenic parental strains were grown for five hours in minimal medium (GYNB), these exponential cells were washed and incubated with the cultured epithelial cells for 1 h, after which the nonadherent fungal cells were washed off, and those adhering to the epithelial cells were then quantified (Materials and Methods). When the data from five independent experiments were combined, the adherence of *xog1* and *eng1* cells was found to be significantly lower than the corresponding parental controls, and this was the case for both the vaginal and colon cell lines (Fig. 5A and B, respectively). This was consistent with a previous report indicating that deleting *XOG1* reduces the adhesion of *C. albicans* to an abiotic polystyrene surface (Tsai et al., 2011a). We conclude that both the Xog1 exoglucanase and the Eng1 endoglucanase promote the adherence of *C. albicans* cells to host epithelial cells.

### 3.5 Influence of Xog1 and Eng1 during systemic candidiasis

**Fig. 6:**
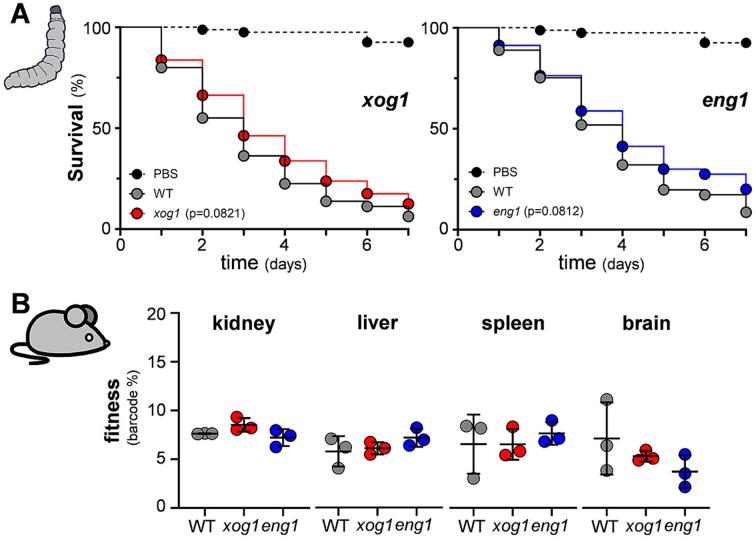
Virulence of *C. albicans xog1* and *eng1* cells during systemic infection. (A**)** Virulence in the *Galleria* model of systemic infection. *C. albicans* strains were grown in GYNB, harvested during exponential growth, washed, and resuspended in PBS: *xog1* B07, *xog1* B08, *xog1* B11, *xog1* C10, *eng1* C01, *eng1* C08, *eng1* C09, *eng1* D08, WT B07, WT B08, WT B11, WT C10, WT C01, WT C08, WT C09 and WT D08 (Supplementary Table S1). About 1 x 10^5^ cells of each strain were injected into 20 *Galleria* larvae, and their survival monitored over eight days. In parallel, as a control, 20 larvae were injected with carrier alone (PBS). The data for the *xog1* mutants and their corresponding parental strains were combined (left panel), as were those for the *eng1* mutants and their parents (right panel). The data were analysed using the log-rank Mantel-Cox test. (B**)** The fitness of barcoded *xog1, eng1* and wild type strains were compared directly in a mouse model of systemic candidiasis: *xog1* B07, *xog1* B08, *xog1* B11, *xog1* C10, *eng1* C01, *eng1* C08, *eng1* C09, *eng1* D08, WT A03, WT A10, WT A11, and WT B04 (Supplementary Table S1). The *C. albicans* strains were grown separately overnight, regrown for 5 h in GYNB, washed, and resuspended in PBS. The strains were then pooled together and injected into the tail veins of three female BALB/C mice. Tissues were harvested after two days, and the relative level of each strain in the kidney, liver, spleen and brain measured by barcode sequencing. Each dot represents the mean for the four independent *xog1* (red)*, eng1* (blue) or wild type control strains (grey) in the given tissue for a single mouse. Means and standard deviations are shown for these mice. The data were analysed using one-way ANOVA: no differences were statistically significant at *p* > 0.05.

We then tested the influence of Xog1 and Eng1 on the virulence of *C. albicans* during systemic infection*,* initially using the *Galleria mellonella* invertebrate model. Once again, all of the independent *xog1* and *eng1* mutants and their parental controls were examined in these experiments (Supplementary Table S1). *Galleria* larvae were injected with a single strain (n = 20 per strain) and their survival monitored over the following days. Those inoculated with the parental strains were killed slightly faster than those infected with the *xog1* and *eng1* mutants, but these differences were not statistically significant (*p* = 0.08: Fig. 6A).

We then compared the relative fitness of the *xog1* and *eng1* mutants directly in a murine model of systemic candidiasis. To distinguish mutants from wild type strains, isogenic wild type controls carrying different barcodes were used in these experiments (WT A03; WT A10; WT A11; WT B04: Supplementary Table S1). All four barcoded *xog1* and *eng1* mutants and the wild type controls were grown separately in GYNB and washed. Then approximately equal proportions of each strain were pooled together, and this mixed population was injected into the tail veins of three mice. After two days, the mice were sacrificed, and their tissues removed. Barcoded sequencing was then performed to quantify the relative proportions of each strain in the kidneys, brains, livers and spleens of these mice. No significant differences between the *xog1* and *eng1* mutants and the wild type controls were found with respect to their fitness in the kidney, brain, liver or spleen (Fig. 6B). Therefore, under the conditions we examined, Xog1 and Eng1 do not appear to make major contributions to the virulence of *C. albicans* during systemic candidiasis.

### 3.6 Influence of Xog1 and Eng1 during gut colonisation

**Fig. 7:**
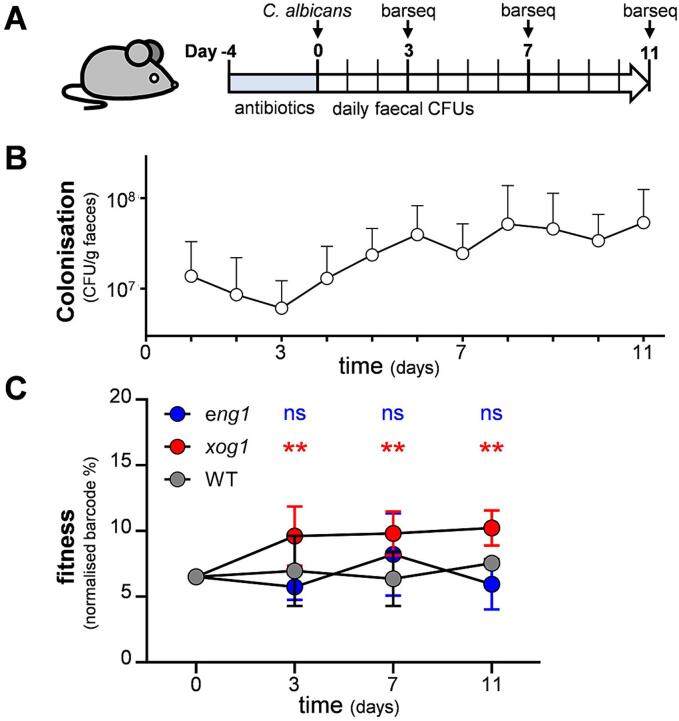
Fitness of *C. albicans xog1* and *eng1* cells during gut colonisation. (A) Cartoon representing the experimental design. Barcoded *C. albicans xog1, eng1* and wild type strains were compared directly in a mouse model of gut colonisation: *xog1* B07, *xog1* B08, *xog1* B11, *xog1* C10, *eng1* C01, *eng1* C08, *eng1* C09, *eng1* D08, WT A03, WT A10, WT A11, and WT B04 (Supplementary Table S1). Each *C. albicans* strain was grown separately in GYNB, resuspended in PBS and then mixed with the others to generate a mixed population containing approximately equal amounts of each strain. This mixed population was then used to gavage six antibiotic treated female BALB/C mice. Fungal burdens (faecal CFUs) were then monitored daily (B), and faecal samples were retained at 3, 7 and 11 days for barcode sequencing along with the initial mixed inoculum. The relative proportion of each strain at each timepoint was normalised to their proportion in the initial inoculum, and then the means and standard deviations for the four independent *xog1* (red)*, eng1* (blue) and wild type control strains (grey) were plotted at each timepoint (C). The data were analysed by two-way ANOVA: ns, not significant; * *p <* 0.05; ** *p <* 0.01.

β-1,3-glucan exposure is thought to compromise the ability of *C. albicans* to colonise the gastrointestinal tract (Sem et al., 2016), and β-1,3-glucan masking appears to promote colonisation of the colon (Avelar et al., 2022). Therefore, we investigated the relative fitness of the *eng1* and *xog1* mutants in the murine gut. Briefly, four barcoded *eng1, xog1* and wild type strains were grown separately on GYNB, these twelve strains pooled, and this mixed population was used to gavage eight antibiotic-treated mice. Fungal colonisation was then monitored over the next eleven days, and samples were taken for barcode sequencing at days zero (input population), three, seven and eleven (Fig. 7A). Fungal colonisation slowly increased in the guts of the eight mice over the course of the experiment (Fig. 7B). Over this period, the *eng1* and wild type strains remained at similar proportions to their starting levels in the input population (Fig. 7C). Meanwhile, there was a small, but significant increase in the relative abundance of the *xog1* strains, suggesting that the inactivation of Xog1, but not Eng1, renders *C. albicans* slightly more fit in this model of gastrointestinal colonisation.

### 3.7 Influence of Xog1 and Eng1 during vaginal colonisation

Host recognition of fungal PAMPs is thought to drive the hyperinflammation that underlies vulvovaginal candidiasis (Fidel et al., 2004, Rosati et al., 2020, Yano et al., 2018). Furthermore, a *STE*11^Δ^*^N467^* signalling mutant that induces β-1,3-glucan unmasking alters immunopathological biomarkers in a murine model of vulvovaginal candidiasis (Wagner et al., 2022). Therefore, we reasoned that Eng1, and potentially Xog1, might influence responses to *C. albicans* in the vagina.

**Fig. 8:**
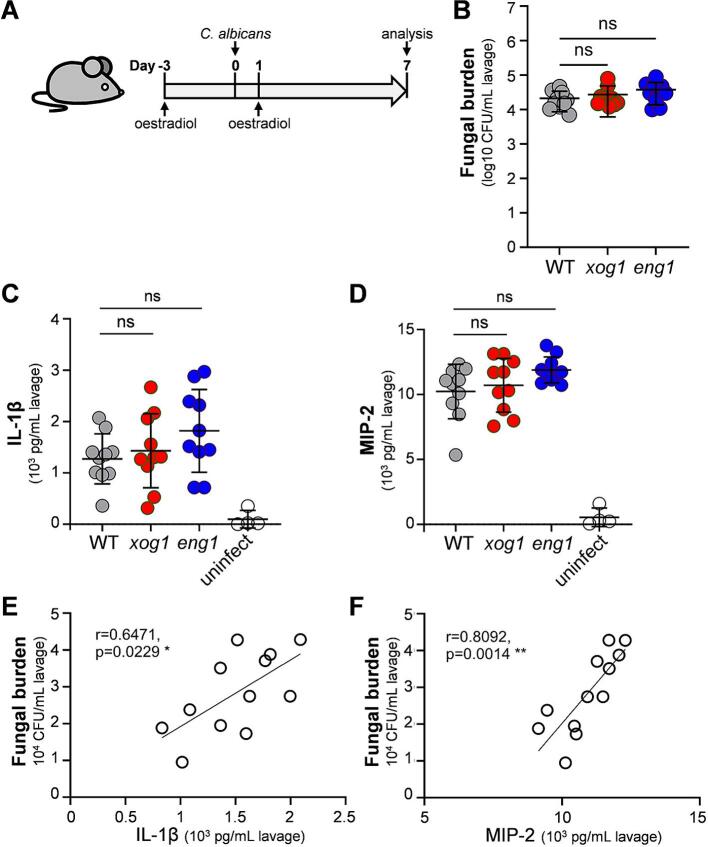
Impact of Xog1 and Eng1 on vaginal colonisation by *C. albicans*. (A) Cartoon representing the experimental design. *C. albicans xog1* (red)*, eng1* (blue) and wild type strains (grey) were compared during vaginal colonisation in oestradiol treated BALB/C mice: *xog1* B07, *xog1* B08, *xog1* B11, *xog1* C10, *eng1* C01, *eng1* C08, *eng1* C09, *eng1* D08, WT A03 WT A10, WT*A11* and WT B04 (Supplementary Table S1). The *C. albicans* cells were grown in GYNB, resuspended in PBS and about 2 x 10^7^ inoculated intravaginally into the mice (n = 3 per barcoded strain). In parallel, as a control, 4 uninfected mice were examined. Vaginal lavage was collected on day 7 post-infection, and in the infected mice fungal burdens measured by counting CFUs (B). The levels of IL-1β (C) and MIP-2 (D) were assayed in each vaginal lavage, including the uninfected control mice (white). Each dot in B, C and D represents data from a single mouse. Means and standard deviations are shown, and the data were analysed by one-way ANOVA: ns, not significant, *p* > 0.05. The correlations between the fungal burden and IL-1β (E) or MIP-2 (F) in each mouse were examined. The r value and statistical significance of each correlation are presented on each plot: * *p <* 0.05; ** *p <* 0.01.

To test this, we turned again to our isogenic set of *eng1, xog1* and wild type control strains (Supplementary Table S1). However, Wagner and coworkers found that the levels of *C. albicans* colonisation in the murine vagina were not affected by *STE*11^Δ^*^N467^*-mediated β-1,3-glucan unmasking (Wagner et al., 2022). Therefore, rather than comparing the *eng1, xog1* and wild type strains in parallel by barcoding, we examined the strains separately. Following treatment with oestradiol, mice were infected intravaginally with the *C. albicans* strains (three mice for each independent mutant; twelve mice in total for each genotype: Fig. 8A), and the mice examined after seven days. Four uninfected mice were included in the study as a control. No significant differences between the *eng1, xog1* and wild type control strains were observed with respect to their fungal burdens in the vagina (Fig. 8B), which was consistent with the findings of Wagner and coworkers (Wagner et al., 2022). We examined the vaginal concentrations of the proinflammatory IL-1β cytokine and MIP-2 chemokine in the infected mice. We selected these for analysis because IL-1β and MIP-2 have been used as readouts for inflammation in the murine model of vulvovaginal candidiasis (Roselletti et al., 2023, Saavedra et al., 1999). IL-1β and MIP-2 levels were increased in the infected animals, but they were not significantly different between mice infected with *eng1, xog1* or wild type *C. albicans* (Fig. 8C and D). There was variability between individual mice with respect to their fungal burdens and IL-1β and MIP-2 levels. Nevertheless, the levels of IL-1β and MIP-2 correlated with fungal burden (Fig. 8E and F).

## 4 Discussion

Three important conclusions arise from this study about the impacts of the exoglucanase Eng1 and the major endoglucanase Xog1 upon cell surface-related phenotypes in *C. albicans*. Firstly, both the endoglucanase Eng1 and the major exoglucanase Xog1 enhance the adhesion of *C. albicans* to epithelial cells, but Eng1 plays a greater role than Xog1 in promoting lactate-induced β-1,3-glucan masking. Secondly, the strength of the latter phenotype can be influenced by confounding factors, at least some of which are water-based, and this raises the question as to the impact of lactate-induced β-1,3-glucan masking within host niches. Nevertheless, thirdly, Eng1 and Xog1 appear to exert subtle effects upon *C. albicans* fitness during infection.

Lactate-induced β-1,3-glucan “masking” is a complex phenotype that is influenced by: (a) the “shielding” of most β-1,3-glucan in the inner cell wall layer by the outer layer of mannan fibrils (Lenardon et al., 2020, Wheeler and Fink, 2006); (b) the “exposure” of β-1,3-glucan at new bud scars during cell division (de Assis et al., 2022), and (c) the lactate-induced “shaving” of exposed β-1,3-glucan by secreted glucanases (Childers et al., 2020a). Therefore, it is hardly surprising that the lactate-induced β-1,3-glucan masking phenotype is influenced by other factors in addition to lactate, which no doubt accounts for the variation in the degree of masking observed for wild type cells between experiments (Fig. 2D and Supplementary Fig. 4). Indeed, we recently reported a modest correlation between growth and β-1,3-glucan exposure (Avelar et al., 2024). Therefore, subtle inputs that influence growth (such as the physicochemical properties of water: Supplementary Fig. 1) may influence lactate-induced β-1,3-glucan masking. This suggests that other mechanisms are likely to contribute to the significant impact of lactate upon *C. albicans* virulence (Fig. 1). These might include enhanced stress resistance, for example (Ene et al., 2012).

Several observations suggested a potential role for Xog1 in the lactate-induced shaving of exposed β-1,3-glucan from the cell surface (Childers et al., 2020a). Firstly, culture medium harvested from masked cell populations can induce β-1,3-glucan masking on unmasked *C. albicans* cells (Childers et al., 2020a), suggesting that a secreted enzyme(s) can shave exposed β-1,3-glucan from the cell surface. Secondly, Xog1 is the major secreted exoglucanase in *C. albicans* (Del Mar González et al., 1997)*,* and the deletion of *XOG1* appeared to attenuate lactate-induced β-1,3-glucan masking (Childers et al., 2020a). Thirdly, the levels of Xog1 (and Eng1) in the secretome increase in response to lactate (12.4- and 2.4-fold, respectively) (Childers et al., 2020a). However, our re-examination of Xog1 has suggested that this major exoglucanase does not make a major contribution to lactate-induced β-1,3-glucan shaving, at least under the conditions we examined in this study (Fig. 2D). On the other hand, the endoglucanase Eng1 does contribute to β-1,3-glucan shaving, although *ENG1* deletion does not completely block shaving (Fig. 2D). This implicates additional factors in lactate-induced β-1,3-glucan masking.

Our previous analyses revealed that lactate enhances the levels of other β-glucan-related enzymes in the *C. albicans* secretome (Childers et al., 2020a). These include Crh11 (cell wall transglycosylase; 2.0-fold increase), Pga4 (cell surface β-1,3-glucanosyltransferase; 2.2-fold) and Utr2 (cell wall glycosidase; 3.8-fold). We also found that the levels of other cell wall proteins increased in the secretome in response to lactate. These included β-glucan linked cell wall proteins, such as: Als1 (adhesin, 1.7-fold increase); Als4 (adhesin,1.8-fold); Ecm33 (cell wall protein, 1.7-fold); Pir1 (β-glucan linked cell wall protein, 1.9-fold); Rbt5 (cell wall protein, 1.7-fold); Rhd3 (cell wall protein, 2.6-fold); Ssr1 (β-glucan associated cell wall protein, 1.6-fold); Ywp1 (yeast cell wall protein: 1.9-fold); and Cht2 (chitinase: 2.0-fold) (Childers et al., 2020a). Significantly, Cottier and coworkers found that *CHT2* influences the exposure of chitin, another major cell wall PAMP, in response to ambient pH (Cottier et al., 2019)*.* They also found that “*a small, heat-stable, nonproteinaceous secreted molecule(s)*” was involved in chitin masking (Cottier et al., 2019), thereby raising the possibility that non-enzymic mechanisms might also contribute to lactate-induced β-1,3-glucan masking. Therefore, this phenotype might involve subtle changes in mannan masking and changes in β-glucan crosslinking, as well as shaving by glucanases.

To what extent do the Xog1 and Eng1 glucanases influence the ability of *C. albicans* to colonise or infect the host? Given the reported impacts of Eng1 and Xog1 upon lactate-induced β-1,3-glucan masking (Childers et al., 2020a, Yang et al., 2022), and given that lactate exposure enhances the virulence of *C. albicans* during systemic infection (Ene et al., 2012) (Fig. 1), we had expected *eng1* and *xog1* mutants to display slight reductions in fitness *in vivo*. This expectation seemed to be borne out, albeit on the edge of statistical significance, in an invertebrate model of systemic candidiasis (Fig. 6A). However, our direct comparison of isogenic *eng1, xog1* and wild type strains in a murine model suggested that the inactivation of Xog1 or Eng1 did not significantly influence the fitness of *C. albicans* in the kidneys, liver, spleen or liver of the female BALB/C mice we examined (Fig. 6B). Yang and coworkers, who reported that an *eng1* mutant displays increased virulence during systemic infections in female C57BL/6 mice (Yang et al., 2022), and this effect was more dramatic in their longer-term virulence model (30 days versus our two-day model). However, these authors observed reduced virulence for the same *eng1* strain in male mice (Yang et al., 2022). Meanwhile, Gonzalez and coworkers reported that mice display longer mean survival times when infected with *C. albicans xog1/xog1-URA3* cells relative to a *C. albicans* SC5314 control (Del Mar González et al., 1997). However, this difference could conceivably have been due to *URA3* position effects as these are known to compromise *C. albicans* virulence (Brand et al., 2004). Taken together, the data suggest that Eng1, and possibly Xog1, might under certain circumstances influence *C. albicans* virulence during systemic infection. If so, such effects could conceivably contribute to the enhanced virulence of *C. albicans* following lactate exposure (Ene et al., 2012) (Fig. 1).

Regarding gut colonisation, Gpr1/Gpa2-mediated changes in β-1,3-glucan exposure appear to correlate with colonisation of the colon (Avelar et al., 2022). Gpr1 is related to the lactate receptor in mammalian cells (Ballou et al., 2016). Also, the inactivation of this G-protein coupled receptor plus its G-protein alpha subunit, Gpa2, blocks lactate-induced β-1,3-glucan masking (Ballou et al., 2016). Taken together, these observations suggested that this phenotype might promote gut colonisation. However, Gpr1 and Gpa2 are also required for β-1,3-glucan exposure in response to another gut fermentation acid, butyrate (Avelar et al., 2022). Furthermore, Gpr1 and Gpa2 regulate yeast-hypha morphogenesis in *C. albicans* (Maidan et al., 2005)*.* Therefore, the effects of Gpr1 and Gpa2 inactivation upon gut colonisation could be mediated by mechanisms other than lactate-induced β-1,3-glucan masking. Indeed, this would be consistent with our observation that the fitness of *C. albicans* in the gut is not compromised by the inactivation of Eng1 (or Xog1) (Fig. 7).

The observation that *xog1* cells displayed significantly enhanced fitness in the gut is intriguing (Fig. 7). Xog1 has been shown to bind the antimicrobial peptides LL-37 and hBD-3 (Chang et al., 2012), and LL-37 binding to *C. albicans* cells reduces their ability to adhere to mammalian cells (Tsai et al., 2011a, Tsai et al., 2011b). Therefore, it is conceivable that *XOG1* inactivation enhances *C. albicans* fitness by compromising some impacts of antimicrobial peptides.

During vulvovaginal candidiasis, β-1,3-glucan becomes exposed on *C. albicans* cells as innate immune cells strip the mannan outer layer from the fungal cell surface (Pericolini et al., 2018). However, the unmasking of β-1,3-glucan at the *C. albicans* cell surface, albeit via a *STE*11^Δ^*^N467^* mutation, does not affect fungal colonisation of the murine vagina (Wagner et al., 2022). Consistent with this, our *eng1* and *xog1* mutants displayed similar levels of vaginal colonisation to their isogenic wild type controls (Fig. 8B). In contrast to the *STE*11^Δ^*^N467^* mutant (Wagner et al., 2022), the *eng1* and *xog1* mutants did not enhance inflammation significantly in the vagina (Fig. 8C and 8D). No doubt the modest effects of the *xog1* and *eng1* mutants upon β-1,3-glucan exposure (Fig. 2C) compared to that for the *STE*11^Δ^*^N467^* mutant (Chen et al., 2019) account for this.

While the impact of Eng1 and Xog1 upon lactate-induced β-1,3-glucan masking is not as strong as we anticipated, this does not detract from the significant impact of β-1,3-glucan masking in general upon host recognition and infection. Other host inputs such as ambient pH, hypoxia and iron limitation significantly affect β-1,3-glucan exposure at the *C. albicans* cell surface, and this influences the ability of innate immune cells to respond to the fungus (Ballou et al., 2016, Childers et al., 2020a, Cottier et al., 2019, Pradhan et al., 2018, Pradhan et al., 2019, Sherrington et al., 2017). Consequently, as *C. albicans* adapts to host inputs it becomes a moving target for the immune system (Brown et al., 2014) making the fungus more difficult to clear from infection sites (Lopes et al., 2018). Further exploration of the factors that contribute to fungal PAMP masking is required to gain a deep understanding of host-fungus interactions during infection.

## CRediT authorship contribution statement

**Qinxi Ma:** Writing – review & editing, Methodology, Investigation, Formal analysis. **Arnab Pradhan:** Writing – review & editing, Methodology, Investigation, Formal analysis. **Ian Leaves:** Writing – review & editing, Methodology, Investigation. **Emer Hickey:** Writing – review & editing, Methodology, Investigation. **Elena Roselletti:** Writing – review & editing, Methodology. **Ivy Dambuza:** Writing – review & editing, Methodology. **Daniel E. Larcombe:** Writing – review & editing, Methodology. **Leandro Jose de Assis:** Writing – review & editing, Methodology. **Duncan Wilson:** Writing – review & editing, Supervision, Methodology. **Lars P. Erwig:** Writing – review & editing, Funding acquisition. **Mihai G. Netea:** Writing – review & editing, Funding acquisition. **Delma S. Childers:** Writing – review & editing, Validation, Conceptualization. **Gordon D. Brown:** Writing – review & editing, Resources, Methodology, Funding acquisition. **Neil A.R. Gow:** Writing – review & editing, Supervision, Funding acquisition, Conceptualization. **Alistair J.P. Brown:** Writing – review & editing, Writing – original draft, Visualization, Supervision, Project administration, Conceptualization.

## Declaration of competing interest

The author declares the following financial interests/personal relationships which may be considered as potential competing interests: NARG is a senior editor of *The Cell Surface* but took no part in the review process. The authors declare that they have no other competing interests or personal relationships that could have appeared to influence the work reported in this paper.
