# Supplementary material for: Impact of secreted glucanases upon the cell surface and fitness of Candida albicans during colonisation and infection
Source: Cell Surf. 2024 Jun 4;11:100128. doi: 10.1016/j.tcsw.2024.100128 (PMC11208952; doi:10.1016/j.tcsw.2024.100128)
Supplement: Supplementary Data 1 [file mmc1.pdf]

Supplementary Figure S1

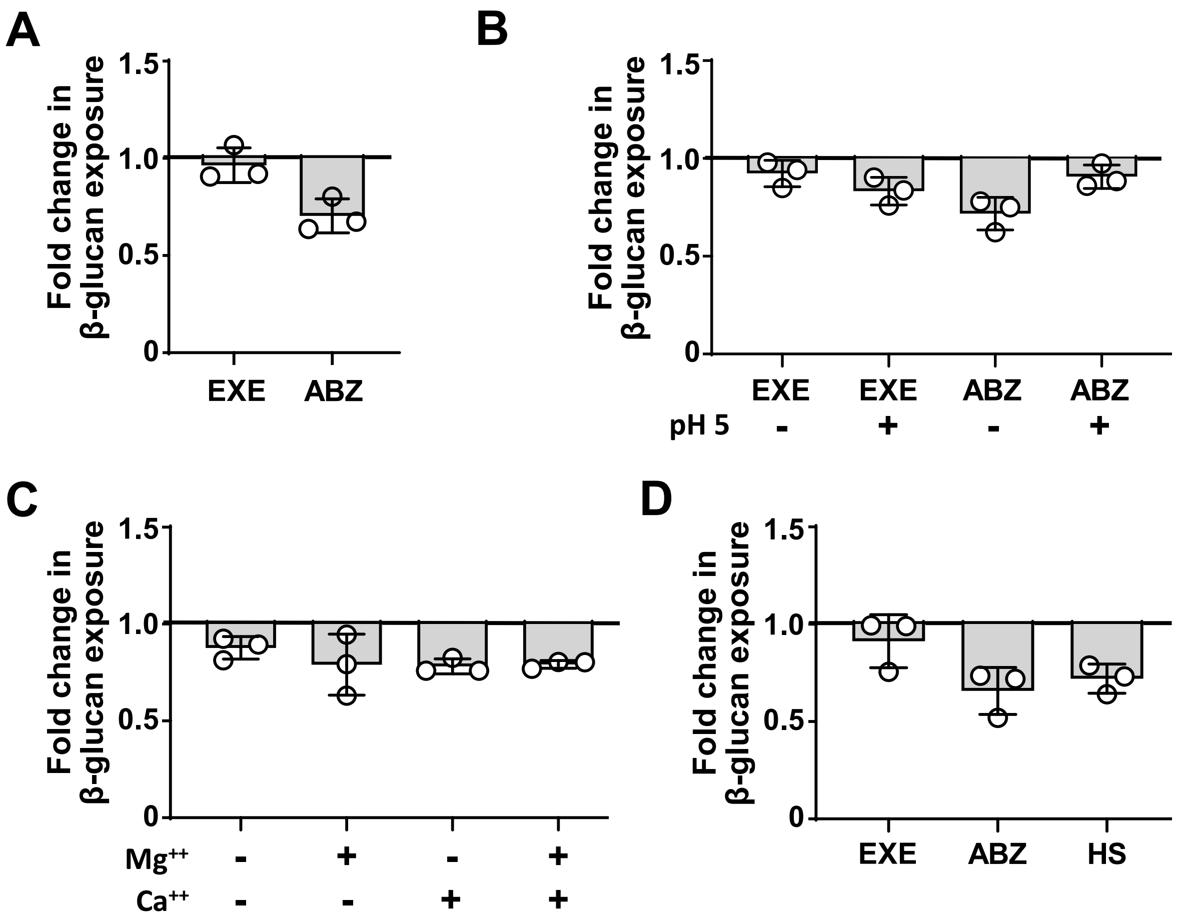

Supplementary Fig. S1. Water influences lactate-induced β-1,3-glucan masking. Lactate-induced β-1,3-glucan masking was assayed using *C. albicans* SC5314 cells grown in GYNB media (Materials and Methods) made using different water sources or with different additives to the water. A. Lactate-induced β-1,3-glucan masking in Milli-Q water from our new laboratory at the University of Exeter (EXE) versus our old laboratory at the University of Aberdeen (ABZ). B. Effect upon lactate-induced β-1,3-glucan masking of adjusting the pH to 5 upon masking in Milli-Q water from Exeter (EXE) versus Aberdeen (ABZ). C. Effect upon lactate-induced β-1,3-glucan masking of adding concentrations of Ca^{++} (1 mM) or Mg^{++} (5 mM) approximately equivalent to those found in hard water to the Milli-Q water from Aberdeen (ABZ). D. Comparison on lactate-induced β-1,3-glucan masking in Milli-Q water from Exeter (EXE), Milli-Q water from Aberdeen (ABZ) or Highland Spring water (HS).
