# Supplementary material for: Impact of secreted glucanases upon the cell surface and fitness of Candida albicans during colonisation and infection
Source: Cell Surf. 2024 Jun 4;11:100128. doi: 10.1016/j.tcsw.2024.100128 (PMC11208952; doi:10.1016/j.tcsw.2024.100128)
Supplement: Supplementary Data 2 [file mmc2.pdf]

Supplementary Figure S2

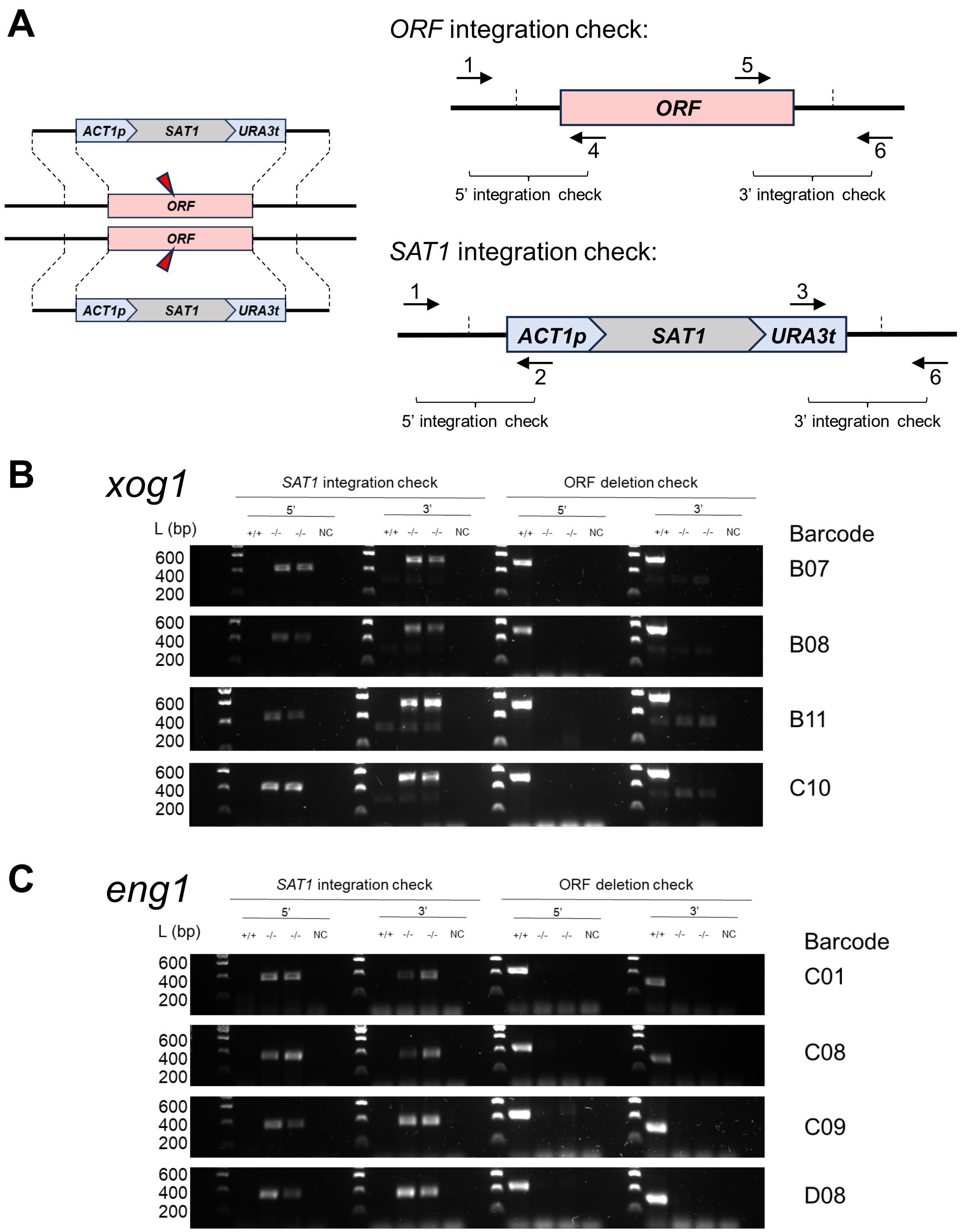

Supplementary Fig. S2. Construction and genotyping of barcoded *C. albicans xog1* and *eng1* mutants. A. Strategy for the mutant construction via CRISPR showing the positions of the diagnostic primers (Supplementary Table S2). B. Genotyping by diagnostic PCR to confirm the integration of the *SAT1* marker and concomitant deletion of the open reading frame at the *XOG1* locus in the four barcoded *xog1* mutants: *xog1*Δ B07, *xog1*Δ B08, *xog1*Δ B11, *xog1*Δ C10 (Supplementary Table S1). C. Corresponding genotyping of the four barcoded *eng1* mutants: *eng1*Δ C01, *eng1*Δ C08, *eng1*Δ C09, *eng1*Δ D08 (Supplementary Table S1).
