# Supplementary material for: Impact of secreted glucanases upon the cell surface and fitness of Candida albicans during colonisation and infection
Source: Cell Surf. 2024 Jun 4;11:100128. doi: 10.1016/j.tcsw.2024.100128 (PMC11208952; doi:10.1016/j.tcsw.2024.100128)
Supplement: Supplementary Data 3 [file mmc3.pdf]

Supplementary Figure S3

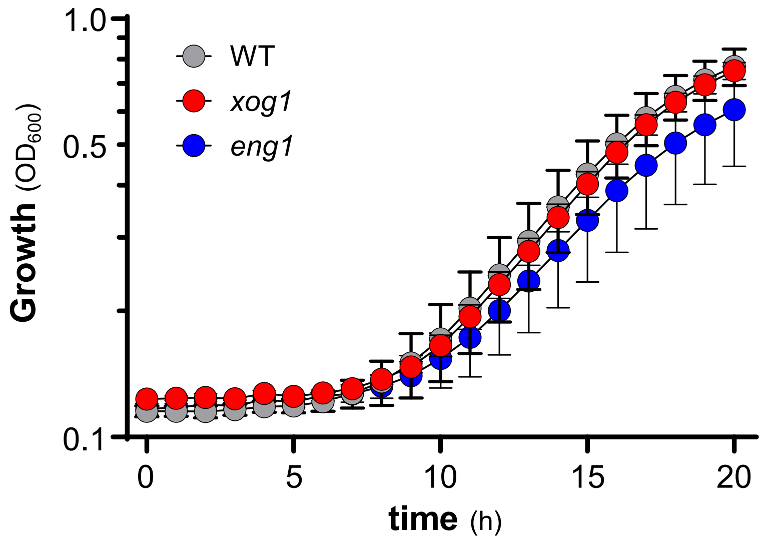

Supplementary Fig. S3. Growth of the *C. albicans xog1* and *eng1* mutants. The growth (OD_{600}) of the four independent *xog1* strains (*xog1*Δ B07, *xog1*Δ B08, *xog1*Δ B11, *xog1*Δ C10), four *eng1* mutants (*eng1*Δ C01, *eng1*Δ C08, *eng1*Δ C09, *eng1*Δ D08), and four wild type controls (WT A03, WT A10, WT A11, WT B04) (Supplementary Table S1) were compared in GYNB at 30 °C with shaking at 200 rpm. The means and standard deviations for the four independent replicates are presented: *xog1*, red; *eng1,* blue; wild type control, grey.
