# Supplementary material for: Impact of secreted glucanases upon the cell surface and fitness of Candida albicans during colonisation and infection
Source: Cell Surf. 2024 Jun 4;11:100128. doi: 10.1016/j.tcsw.2024.100128 (PMC11208952; doi:10.1016/j.tcsw.2024.100128)
Supplement: Supplementary Data 4 [file mmc4.pdf]

Supplementary Figure S4

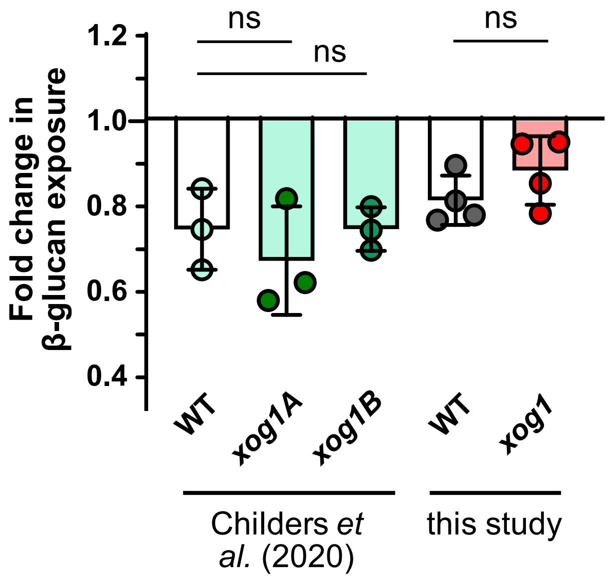

Supplementary Fig. S4. Comparison of lactate-induced β-1,3-glucan masking in different *C. albicans xog1* mutants. Lactate-induced β-1,3-glucan masking was compared in the two independent null mutants (*xog1A, xog1B,* green) made from its wild type parent SC5314 (WT, pale green) as described by Childers and co-workers [Childers et al. (2020) *mBio* 11, e00984-20], and in the four independent mutants (*xog1*Δ B07, *xog1*Δ B08, *xog1*Δ B11, *xog1*Δ C10, red) and their parents (WT B07, WT B08, WT B11, WT C10, dark grey) from this study (Supplementary Table S1). Data for the former strains represents means and standard deviations from three independent replicates. Data for the latter strains represents means and standard deviations from combining single measurements for each of the four barcoded strains; ns, not significant, $p > 0.05$.
