# Supplementary material for: Impact of secreted glucanases upon the cell surface and fitness of Candida albicans during colonisation and infection
Source: Cell Surf. 2024 Jun 4;11:100128. doi: 10.1016/j.tcsw.2024.100128 (PMC11208952; doi:10.1016/j.tcsw.2024.100128)
Supplement: Supplementary Data 5 [file mmc5.pdf]

Supplementary Figure S5

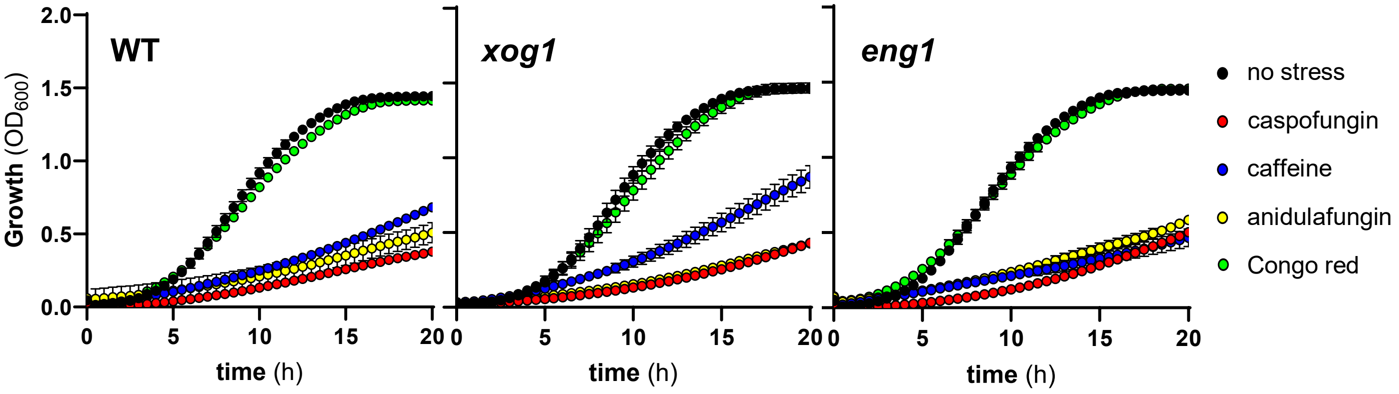

Supplementary Fig. S5. Cell wall stress resistance of *C. albicans xog1* and *eng1* mutants in liquid medium. *C. albicans* wild type, *xog1* and *eng1* strains were inoculated into 96-well plates with YPD containing different cell wall stressors, and their growth monitored over time (OD_{600}): 62.5 ug/ml Congo Red (green symbols); 10 mM caffeine (blue); 0.1 ug/ml caspofungin (red); 0.05 ug/ml anidulafungin (yellow); no stress control (black). Each data point shows the mean and standard deviation for 3 technical replicates from 3 independent wild type, *xog1* or *eng1* strains (9 replicates in total).
