# Supplementary material for: Impact of secreted glucanases upon the cell surface and fitness of Candida albicans during colonisation and infection
Source: Cell Surf. 2024 Jun 4;11:100128. doi: 10.1016/j.tcsw.2024.100128 (PMC11208952; doi:10.1016/j.tcsw.2024.100128)
Supplement: Supplementary Data 6 [file mmc6.pdf]

Supplementary Table S1. *C. albicans* strains used in this study

Strain	Name	Parent	Genotype	Reference/Source
SC5314	SC5314	-	blood isolate	1
CAI4	CAI4	SC5314	*ura3Δ::imm434/Δura3Δ::imm434*	2
Ca 2390	*xog1*Δ A	SC5314	*xog1*Δ::*FRT*/*xog1*Δ::*FRT*	3
Ca 2391	*xog1*Δ B	SC5314	*xog1*Δ::*FRT*/*xog1*Δ::*FRT*	3
barcoded strains		CAI4	CAI4, *RPS1*-CIp10-Ptet-GTw (URA3)	below

**BARCODED *C. ALBICANS* STRAINS**				
Genotype: CAI4, *RPS1-CIp10-pTET-GW (URA3)*				
**Strain**		**Barcode**	**Barcode sequence** (common 5' sequence-**unique barcode**-common 3' sequence)	**Source**
Ca 2247	WT A03	BC_A03	CGGTGTCGGTCTCGTAG**TACAATAAAGGGCAGGTGCAACCAT**AGAGACCTCGTGGACATC	4
Ca 2249	WT A08	BC_A08	CGGTGTCGGTCTCGTAG**CACAGACGACGTAACTTTTATAGGC**AGAGACCTCGTGGACATC	4
Ca 2251	WT A10	BC_A10	CGGTGTCGGTCTCGTAG**TGACCAAGCCTGAATAGCGTCATAA**AGAGACCTCGTGGACATC	4
Ca 2253	WT A11	BC_A11	CGGTGTCGGTCTCGTAG**CGTGTATTAGAGTAATCGCATCTAG**AGAGACCTCGTGGACATC	4
Ca 2255	WT A12	BC_A12	CGGTGTCGGTCTCGTAG**ACGAAGCGTGAAACGTATCGAACGC**AGAGACCTCGTGGACATC	4
Ca 2257	WT B04	BC_B04	CGGTGTCGGTCTCGTAG**TAGCGTTAGCGGCCACGGAACATCA**AGAGACCTCGTGGACATC	4
Ca 2259	WT B05	BC_B05	CGGTGTCGGTCTCGTAG**TTAATTGCGGGCAGCCACCGTGAAT**AGAGACCTCGTGGACATC	4
Ca 2261	WT B07	BC_B07	CGGTGTCGGTCTCGTAG**TCCTCTCGGGAGGCCAATAGAAACG**AGAGACCTCGTGGACATC	4
Ca 2263	WT B08	BC_B08	CGGTGTCGGTCTCGTAG**TACGCTTGACCCGTGTAGCTGTCAG**AGAGACCTCGTGGACATC	4
Ca 2265	WT B11	BC_B11	CGGTGTCGGTCTCGTAG**TGGTTAACACGGAACCTGAGATCCC**AGAGACCTCGTGGACATC	4
Ca 2267	WT C10	BC_C10	CGGTGTCGGTCTCGTAG**TAGCACACCTAGCGTGAACCGAGCC**AGAGACCTCGTGGACATC	4
Ca 2277	WT C01	BC_C01	CGGTGTCGGTCTCGTAG**TTACCCATTAAGAGCTCAGCGCAAC**AGAGACCTCGTGGACATC	This study
Ca 2280	WT C08	BC_C08	CGGTGTCGGTCTCGTAG**TCCGTGTTGTACGACGTTAGACAAC**AGAGACCTCGTGGACATC	This study
Ca 2281	WT C09	BC_C09	CGGTGTCGGTCTCGTAG**TAGTCTACCTCGCATGGCTAAGCGG**AGAGACCTCGTGGACATC	This study
Ca 2282	WT D08	BC_D08	CGGTGTCGGTCTCGTAG**TATCCAGTCGGCGATAAGTATGCAA**AGAGACCTCGTGGACATC	This study
Ca 2609	*xog1*Δ B07	BC_B07	CGGTGTCGGTCTCGTAG**TCCTCTCGGGAGGCCAATAGAAACG**AGAGACCTCGTGGACATC	This study
Ca 2611	*xog1*Δ B08	BC_B08	CGGTGTCGGTCTCGTAG**TACGCTTGACCCGTGTAGCTGTCAG**AGAGACCTCGTGGACATC	This study
Ca 2613	*xog1*Δ B11	BC_B11	CGGTGTCGGTCTCGTAG**TGGTTAACACGGAACCTGAGATCCC**AGAGACCTCGTGGACATC	This study
Ca 2615	*xog1*Δ C10	BC_C10	CGGTGTCGGTCTCGTAG**TAGCACACCTAGCGTGAACCGAGCC**AGAGACCTCGTGGACATC	This study
Ca 2617	*eng1*Δ C01	BC_C01	CGGTGTCGGTCTCGTAG**TTACCCATTAAGAGCTCAGCGCAAC**AGAGACCTCGTGGACATC	This study
Ca 2619	*eng1*Δ C08	BC_C08	CGGTGTCGGTCTCGTAG**TCCGTGTTGTACGACGTTAGACAAC**AGAGACCTCGTGGACATC	This study
Ca 2621	*eng1*Δ C09	BC_C09	CGGTGTCGGTCTCGTAG**TAGTCTACCTCGCATGGCTAAGCGG**AGAGACCTCGTGGACATC	This study
Ca 2623	*eng1*Δ D08	BC_D08	CGGTGTCGGTCTCGTAG**TATCCAGTCGGCGATAAGTATGCAA**AGAGACCTCGTGGACATC	This study

References

1. Gillum AM *et al.* (1984) Isolation of the *Candida albicans* gene for orotidine-5'-phosphate decarboxylase by complementation of *S. cerevisiae ura3* and *E. coli pyrF* mutations. *Molec. Gen. Genet*. 198, 179-182.
2. Fonzi WA & Irwin MY (1993) Isogenic strain construction and gene mapping in Candida albicans. *Genetics* 134, 717-728.
3. Childers, DS *et al.* (2020) Epitope shaving promotes fungal immune evasion. *mBio* 11, e00984-20.
4. Larcombe DE *et al.* (2023) Glucose-enhanced oxidative stress resistance – a protective anticipatory response that enhances the fitness of *Candida albicans* during systemic infection. *PLoS Pathogens* 19, e1011505.
