# Supplementary material for: Impact of secreted glucanases upon the cell surface and fitness of Candida albicans during colonisation and infection
Source: Cell Surf. 2024 Jun 4;11:100128. doi: 10.1016/j.tcsw.2024.100128 (PMC11208952; doi:10.1016/j.tcsw.2024.100128)
Supplement: Supplementary Data 7 [file mmc7.pdf]

Supplementary Table S2. Primers used for the construction of the barcoded *C. albicans* *xog1* and *eng1* mutants

Min K, Ichikawa Y, Woolford CA, Mitchell AP. (2016) *Candida albicans* Gene Deletion with a Transient CRISPR-Cas9 System. mSphere, 1(3):e00130-16.

Primer	Sequence	Description
CaCas9/for	ATCTCATTAGATTTGGAACTTGTGGGTT	Forward and reverse primers for amplification of *CaCas9* cassette
CaCas9/rev	TTCGAGCGTCCCAAAACCTTCT	
SNR52/F	AAGAAAGAAAGAAAACCAGGAGTGAA	Forward primer for amplification of *SNR52* promoter
sgRNA/R	ACAAATATTTAAACTCGGGACCTGG	Reverse primer for amplification of sgRNA scaffold
SNR52/N	GCGGCCGCAAGTGATTAGACT	Forward and reverse nested primers for third round PCR for construction of sgRNA expression cassette
sgRNA/N	GCAGCTCAGTGATTAAGAGTAAAGATGG	

Reverse primer for amplification of *SNR52* promoter with overlapping guide sequence of the target gene
Guide Sequence in red

SNR52-sg-XOG1-Rv	TTGACCTTCAGCAACAGTCACAAATTAAAAATAGTTTACGCAAGTC
SNR52-sg-ENG1-Rv	CAGTTCTTCCAGTTCTGTTGCAAATTAAAAATAGTTTACGCAAGTC

Forward primer for amplification of sgRNA scaffold with overlapping guide sequence of the target gene
Guide Sequence in red

XOG1-sg-scaf-Fw	TGACTGTTGCTGAAGGTCAAGTTTTAGAGCTAGAAATAGCAAGTTAAA
ENG1-sg-scaf-Fw	CAACAGAACTGGAAGAACTGGTTTTAGAGCTAGAAATAGCAAGTTAAA

Primers to amplify the repair template

NAT-XOG1-RT-Fw	TTTCTTTCCCTTCTTTTTTTTTTAAATTGTTCTTCATTACATTTGAATCAAACATATCAAAGATTAATACATAATCAGCTAGTCTAATCACTTGCGGCCGC
NAT-XOG1-RT-Rv	AAGCCTATTTTGAGAAGAAATTTTTTTTAAAATTTTTTTTTTAAAATTATTATTGCTCTTGTAGAATCAAGACGCCTTAAGGACCACCTTTGATTGTAAATAG
NAT-ENG1-RT-Fw	GGTCTGATTCTCAACTTCTACCATTCAATTTATTGAACAAGATTTATCATTCTAGATCATAGTTATACATTAATATATCGCTAGTCTAATCACTTGCGGCCGC
NAT-ENG1-RT-Rv	CCTGATAAAAATAATTATTAAAAACAAAATTAAATGAAAGTGAAATCTTTCACGAAATAAGAATTATAGTACATAAGGGACCACCTTTGATTGTAAATAG

Verfication oligos

NAT-5V-Rv	TATAAATAGCACACACCCAC
NAT-3V-Fw	GTACTGGTACTGGTTCTCGG
XOG1-5V-Fw	CAAACGACAACTTCTTGCAGG
XOG1-ORF5-Rv	CAAGGACAAACCAACCACCC
XOG1-ORF3-Fw	GGTCTGATGAACACAAAACCGAC
XOG1-3V-Rv	CCAGCTTTCAGATTTTATAGTGC
ENG1-5V-Fw	CCGGAATTAGAAGATCCAGC
ENG1-ORF5-Rv	CCAGTAGTTAATCCAGTTGGGTTG
ENG1-ORF3-Fw	GGGATGACAAGTGGTTAGATAATG
ENG1-3V-Rv	CAGTAGTGTCACCTCTTTCAAC
